# The forest environmental frontier in Russia: Between sustainable forest management discourses and ‘wood mining’ practice

**DOI:** 10.1007/s13280-021-01643-6

**Published:** 2021-10-21

**Authors:** Denis Dobrynin, Natalya Yakusheva Jarlebring, Irmeli Mustalahti, Metodi Sotirov, Elena Kulikova, Eugene Lopatin

**Affiliations:** 1 Department of Geographical and Historical Studies, University of Eastern Finland, P.O. Box 111, 80101 Joensuu, Finland; 2 Department of Forest Sciences, University of Helsinki, Latokartanonkaari 7, 00014 Helsinki, Finland; 3 University of Freiburg, Tennenbacher Str. 4, 79106 Freiburg, Germany; 4 European Forest Institute, Yliopistokatu 6B, 80100 Joensuu, Finland; 5 Natural Resources Institute Finland (Luke), Joensuu, Finland

**Keywords:** Forest discourses, Intact forest landscapes, Intensive forest management, Non-state actors, Sustainable forest management, Russia

## Abstract

With 20% of the world’s forests, Russia has global potential in bioeconomy development, biodiversity conservation and climate change mitigation. However, unsustainable forest management based on ‘wood mining’ reduces this potential. Based on document analysis, participant observations and interviews, this article shows how non-state actors—environmental NGOs and forest companies—address forest resource depletion and primary forest loss in Russia. We analyse two key interrelated forest discourses driven by non-state actors in Russia: (1) intensive forest management in secondary forests as a pathway towards sustained yield and primary forest conservation; (2) intact forest landscapes as a priority in primary forest conservation. We illustrate how these discourses have been integrated into policy debates, institutions and practices and discuss their relation to relevant global discourses. The article concludes that despite successful cases in conserving intact forest landscapes, there is still a frontier between sustainable forest management discourses and forestry practice in Russia.

**Supplementary Information:**

The online version contains supplementary material available at 10.1007/s13280-021-01643-6.

## Introduction

The global transition to sustainable development and climate-neutral society in science and policy is associated with the development of a bioeconomy, in particular a forest-based bioeconomy (Pülzl et al. 2014; D'Amato et al. 2017; Holmgren et al. 2020). Harbouring 20% of the world’s forests (FAO 2020), Russia has global potential in forest-based bioeconomy development, as well as in climate change mitigation and biodiversity conservation. However, unsustainable forest management reduces this potential (Newell & Simeone 2014; Leskinen et al. 2020). In Russia, lack of attention to the sustainability of forest management and forest protection is caused by the long-term perception by decision-makers of the forest “as abundant, if not unlimited, resources” (Ulybina and Fennell 2013). The Russian forest sector has relied on so-called ‘wood mining’, where primary forests are harvested, rather than on silviculture in secondary forests based on sustainable forest management principles (Angelstam et al. 2016). As a result of an overestimation of the real harvesting potential within the framework of the government-set annual allowable cut, and also because of fires and pests, the economically available forest resources have been depleted (Elbakidze et al. 2013; Naumov 2017). Forest depletion has rendered the current forestry model based on ‘wood mining’ incapable of meeting the increasing market demand for wood driven by global bioeconomy growth (Leskinen et al. 2020). Researchers and forest professionals are talking about a wood supply crisis in the country (Moiseev 2012; Shvarts and Shmatkov 2020).

Sustainable forest management policy and practices are shaped by the interaction of state regulation and non-state actors (Arts 2006) as well as market-driven tools, such as forest certification (Cashore 2002; Bernstein and Cashore 2004). In Russia, the state forest regulation is implemented by federal agencies and regional governments. Non-state forest actors are environmental NGOs and private forest companies and that entered the forest policy arena in the 1990s after the Soviet Union collapsed (Ulybina 2014). Environmental NGOs operating with sustainable forest management issues are international and domestic professional organizations (Newell and Henry 2017). Private forest companies are transnational corporations and domestic companies that gained forest management rights as concession holders in state-owned forests. In Russia, 80% of the total volume of harvested wood is harvested in forest concessions (The National Strategy for the Development of the Forest Sector until 2030). Thus, the sustainability of forest management in Russia is determined primarily by the sustainability of forestry carried out by private companies in forest concessions. In turn, the sustainability of forest management in forest concessions is determined by state regulation and market-driven forest certification. In Russia, according to Ulybina (2014), forest companies and environmental NGOs collaborate to compensate for the inability of the state to address the problem of unsustainable forest management and to meet market requirements. The Russian forest sector is export-oriented and partly depends on ‘eco-sensitive’ foreign markets. Therefore forest certification schemes, in particular the Forest Stewardship Council (FSC), have become firmly present among the Russian forest governance tools. In Russia, FSC is driven by environmental NGOs as a tool to develop sustainable forest management and primary forest conservation within forest concessions, and by transnational forest companies that need to meet demand on foreign markets, they depend on (Ulybina and Fennell 2013; Henry and Tysiachniouk 2018; Dobrynin et al. 2020). In 2020, Russia became the world leader in certified forests, with 56.6 million hectares (FSC-Russia 2020) representing 27% of the total area of forest concessions in the country (The National Strategy for the Development of the Forest Sector until 2030). In addition, FSC requirements for sustainability of forest management also apply to non-certified forest concessions as part of the requirements on controlled wood.

Despite previous scholarly attention to the study of Russian forest policy and governance (Torniainen et al. 2006; Sotirov and Mashkina 2010; Torniainen et al. 2010; Petrov 2012; Libman & Obydenkova 2014; Ulybina 2014; Tysiachniouk and McDermott 2016), the development of sustainable forest management discourses driven by non-state actors has not yet been well scrutinized in academic literature. In this article, we analyse how non-state actors—environmental NGOs and forest companies—address unsustainable forest management in Russia, including forest resource depletion and primary forest loss. Our interpretative analysis is guided by the following research questions: What key discourses on sustainable forest management and forest conservation are driven by non-state actors in Russia? How are these discourses represented at the level of policy debates (conceptualization), institutions (institutionalization), and practical implementation (materialization)?

Discourses are an important and relevant topic to study as they shape actors’ views and by this, they may result in policy, institutional and behavioural changes (Hajer 1995; Hajer and Versteeg 2005; Arts 2009). The basic assumption of discourse analysis is that language shapes an individual’s view of the world and reality instead of being merely neutral (Hajer and Versteeg 2005). Discourses are both an expression of and a prerequisite for social interaction and practices (Pynnönen 2013; Holmgren 2015). For example, Mustalahti (2018) explains in the context of the forest-based bioeconomy how discourses operate as a key frame of reference and action to define how the world is seen and which ideas can move and be passed on to others.

## Conceptual and analytical framework

### Understanding discourses and governance

In social and political sciences, a discourse is defined as “a specific ensemble of ideas, concepts, and categorizations that are produced, reproduced, and transformed in a particular set of practices and through which meaning is given to physical and social realities” (Hajer 1995, p. 44). Discourses construct meanings and help to define a mutual way of understanding social and natural phenomena (Dryzek 2013). Thus, the underlying assumption of the discourse approach is that new ideas, concepts, paradigms and narratives shape policies, institutions and practices (Arts and Buizer 2009). Discourse analysis is widely applied to examine forest and environmental policy at the international and national level (e.g. Arts et al. 2010; Winkel 2012; Leipold 2014; Mustalahti 2018; Pecurul-Botines et al. 2019). Following Arts et al. (2010), who adapted Hajer’s definition, we understand discourses in forest policy and governance as “a set of (1) ideas, (2) concepts and (3) categorizations that are created and changed in forest-related social practices and which give meaning to forests as both physical and social phenomena”. Arts (2009) notes that discourses reflect conceptualization of the policy problems and the development of solutions.

Taking into account Hajer’s approaches to the analysis of discursive hegemony (Hajer 1995; Hajer and Versteeg 2005), we distinguish conceptualization and institutionalization of the discourses. We understand conceptualization as a discursive stage when a specific idea, or a concept, is structured and becomes well known among policy actors and professionals. Institutionalization of discourses can be seen when the idea, or a concept, is integrated in formal policy documents, regulations and institutions. Moreover, we consider how analysed discourses are represented at the level of materialization—practical implementation in forest management and conservation practices (Arts et al. 2013).

### Forest discourses and governance

According to Kleinschmit et al. (2009) the concept of discourses gained momentum with the development of the debate on new modes of regulation—“governance without government” (Rosenau & Czempiel 1992). The concept of governance in social and political studies since the 1980s implies “governing by, governing with and governing without the state” (Arts and Visseren-Hamdkers 2012, p. 242). Multi-actor and multi-level governance can be understood as the displacement of state power upward to international and intergovernmental organizations, downward to subnational authorities and communities, and outward to private organizations and markets (Pierre and Peters 2000). Forest governance considers how state agencies, non-state actors such as NGOs and private companies, international organizations, and citizens shape and affect forest policymaking and forest management practices through various instruments and coalitions (Agrawal et al. 2008; Sotirov and Arts 2018). We consider the institutionalization and materialization of the analysed discourses on three levels of governance: state regulation, non-state market-driven governance, and intergovernmental structures.

### Global meta, regulatory and forest discourses

**Fig. 1:**
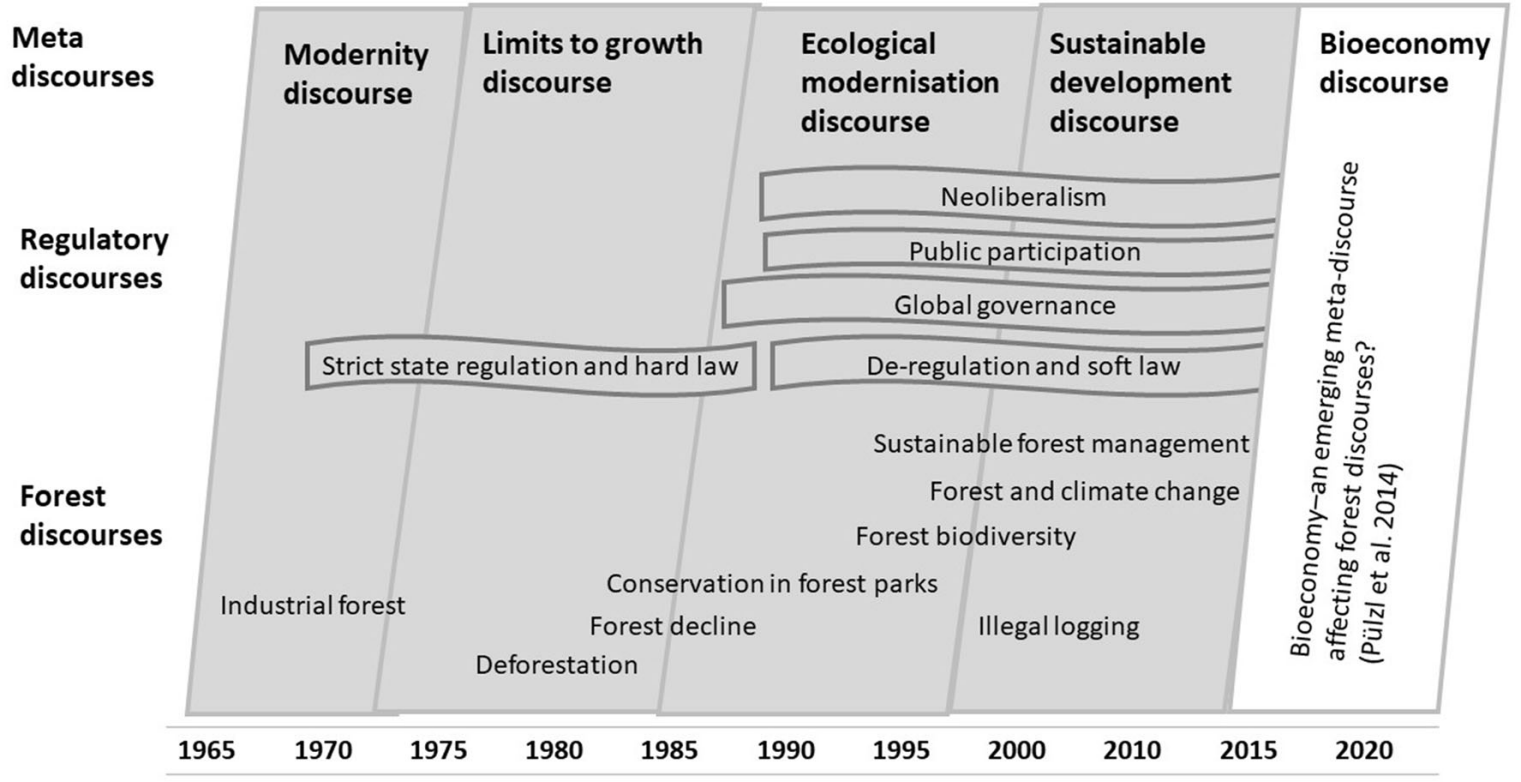
Global meta, regulatory and forest discourses. *Source* adapted and developed from Arts et al. (2010) and Pülzl et al. (2014)

The development of global forest policy is seen in a continuum of meta, regulatory, and forest-related discourses, as suggested by Arts et al. (2010) and updated by Pülzl et al. (2014) (Fig. 1). Global environmental meta-discourses are “modernity”, “limits to growth”, “ecological modernization”, “sustainable development” (Arts et al. 2010), and as of recently “bioeconomy” (Pülzl et al. 2014) (Fig. 1).

Global regulatory discourses are represented by “global governance”, “public participation”, and “neoliberalism”, including the ideas of “de-regulation and soft law” as a replacement for the previously dominating “strict state regulation and hard law” (Arts et al. 2010) (Fig. 1). The aforementioned regulatory discourses are related to privatization and ‘NGO-ization’ of forest governance through voluntary, legally non-binding regulation. Such a type of regulation, also known as “non-state market-driven governance” (Bernstein and Cashore 2004), is embodied in practice in forest certification schemes.

The well-known global forest discourses include “industrial forests”, “deforestation”, “conservation in forest parks”, “forest biodiversity”, forests and climate change”, “illegal logging” and “sustainable forest management” (Arts 2009; Pülzl et al. 2014) (Fig. 1). “Industrial forests” discourse focuses on the forest as a source for economic development, the sustained yield of wood resources and forest concessions in developing countries. “Deforestation” discourse relates to the conversion of tropical forests to agricultural lands, and later this discourse was also related to the decline of temperate and boreal forests. “Conservation in forest parks” discourse concentrates on biodiversity conservation in state protected areas by prohibiting economic activities, sometimes with insufficient attention to the needs of local communities. “Forest biodiversity” discourse is linked with the Convention on Biological Diversity and includes both protection of forest biodiversity for itself and protection of biodiversity-rich forest to mitigate global climate change. “Forests and climate change” discourse is about efforts to reduce carbon emissions from deforestation and forest degradation (REDD), and measures were added later on forest conservation and sustainable management of forests in tropical countries (REDD+). “Illegal logging” discourse is driven by the intergovernmental processes on forest law enforcement and governance (FLEG), with later attention added to encompass trade (FLEGT), to tackle wood from illegal sources in importing countries (Leipold et al. 2016). “Sustainable forest management” discourse reflects the meta-discourse on sustainable development and is understood as a balance of various economic, ecological and socio-cultural interests and values related to forest and forest use. Debates on sustainable forest management includes issues such as sustained yield, biodiversity conservation and actors’ participation, as well as multifunctionality of forestry and integrated forest management (Arts 2009; Sotirov and Arts 2018).

## Materials and methods

The data for this article were collected from participant observations, analysis of literature and documents, and cross-checked through interviews. Participant observations were conducted by the lead author in 2004–2018 through his involvement in NGO activities in Russia, and again in 2018–2020 during his PhD study. In 2004–2018, participant observation which was carried out during meetings, workshops and field trips in Russia constitute the source of the autoethnography materials. Autoethnography implies a critical look at various social beliefs and management practices based on personal experience, self-observation and reflexive exploration, which are used in various research fields (Mosse 2005; Anderson 2006; White 2020). The limitation of the autoethnography approach is that the researcher is not a ‘neutral observer’ but rather an ‘activist insider’, a role which might impact the research process. The researcher should not be drawn into participating in activities at the expense of writing reflective research diaries and carrying out objective research processes (Anderson 2006). For this reason, the lead author stepped out of active involvement in NGO activities in Russia to conduct the research. In 2018–2020, he carried out the second round of participant observations as an observer. This part of data collection as well as analyses of his understanding and reflections with co-authors helped to create a more distant way to analyse the discourses. The lead author's reflections and notes, as well as materials from relevant conferences, seminars, meetings, field workshops and study tours, were used to construct and analyse discourses. Opinion pieces, discussions in online forums, press releases as well as policy documents, government regulations, and certification standards were also sources of data. The analysis of technical and policy documents was supported by scrutiny of relevant academic and grey literature in Russian and English.

The results of the participant observations, document and literature analysis were cross-checked by key informant interviews. The first author conducted five key informant interviews to help interpret the analysed discourse material. These interviews were conducted in Russia in March 2020 as face-to-face meetings, and each interview lasted 1.5–2 h. The key informants were chosen to represent five different fields and included representatives of the forest industry, the state forest inventory service, a forest research institute, a university, and an auditing body conducting forest certification. The informants were asked about the dominant debates on sustainable forest management and forest protection in Russia within the community of forest professionals.

We applied a logic of interpretative analysis (Yanow 2007) to understand the meanings assigned to forest discourses by forest policy actors in Russia. The logic of interpretative analysis implies that meanings of policy and governance issues are context specific. The meanings are derived not only from policy-relevant events and documents but are shaped by the various actors and researchers as meaning-makers themselves (Yanow 2007). Interpretative analysis was carried by a group of authors consisting of ‘insiders’ who have practical experience in Russian forest management and governance issues, and by ‘outsiders’, scholars majoring in forest policy and governance in various regions of the world.

## Results

We have constructed and analysed the following two key interrelated forest discourses driven by non-state actors in Russia: (1) intensive forest management in secondary forests as a pathway towards sustained yield and primary forest conservation; and (2) intact forest landscapes as a priority in primary forest conservation. Key components of the analysed discourses in relation to their conceptualization, institutionalization and materialization are represented in a nutshell in the summary table (Table 1) and in detail in the text below. Examples of relevant citations characterizing the discourses are given in the text below and in Appendix S1.

**Table 1:** Summary table. Key components of the analysed forest discourses in Russia

		Levels of discourses			Key drivers
		Conceptualization	Institutionalization	Materialization	
Discourses	Intensive forest management	A way towards sustainable forest management, based on the ‘Scandinavian’ model of silviculture and sustained yield in secondary forest. A transition from ‘wood mining’ in primary forests towards forestry in secondary forests	– State forest management policies and strategies – State forestry regulations – Company forest management strategies	– Model forests – Company demonstration sites and pilot projects on intensive forest management	NGOs and companies
	Intact forest landscapes	Last large unfragmented primary forest landscapes, shelters of biodiversity and carbon sinks. The priority category of primary forest to be conserved	– FSC forest certification standards – State forest management and conservation strategies	– Non-legally binding moratoria (‘no-go’) zones – Sate Protected areas (national parks and nature reserves)	NGOs

### Intensive forest management discourse: Conceptualization

At the international level, intensive forest management is defined by FAO^1^ as “a regime of forest management, where silvicultural practices define the structure and composition of forest stands” (FAO, 2005). In Russia, NGO-driven debates on sustainable forest management focus on the transition from extensive forest management, also called ‘wood mining’ («*дoбычa дpeвecины»*), in primary forests to intensive forest management (*«интeнcивнoe лecнoe xoзяйcтвo»*) in secondary managed forests, also known as ‘tree agriculture’ («*лecнoй oгopoд»*). The idea of developing intensive forest management has become a key topic of scientific and policy debates related to forests:

The transition from extensive to intensive forest management, from ‘collecting’ to civilized forest cultivation, is the inevitable and the only way for the further development of forest management in the face of increasing demand for wood (Source 1).

This debate has been initiated by environmental NGOs and forest companies:

It is necessary to stimulate intensive forest management in secondary forests to maximize the commercial forest yield through reforestation, forest care, multiple increases in the productivity of leased forests and the volume of investments; representative of an environmental NGO (Source 4).

Intensive forest management for me is a synonym for proper forest management, the management that leads to the entire forestry cycle of operations…; representative of a forest company (Source 2).

In Russia, the transition to sustainable forest management is seen as an application of the Scandinavian (Nordic) model of intensive even-aged (rotation) forestry based on the economic concept of sustained yield (Karjalainen et al. 2009; Naumov et al. 2016; Senko et al. 2018; Sotirov and Arts 2018). This occurs even though the intensive even-aged (rotation) forestry model has been criticized in Scandinavian countries as unsustainable and leading to overexploitation and biodiversity loss (Hanski 2011). Moreover, in Scandinavian countries (and in other European countries) alternative models of sustainable forest management, including continuous cover forestry, close-to-nature forest management or integrated forest management, are currently being developed (Sotirov and Arts 2018; Sotirov and Storch 2018). Similar policy ideas of sustainable continuous cover forestry have not travelled to Russia so far.

In Russia, international environmental NGOs and transnational forest companies (concession holders) have had joint efforts since the 1990s to promote the idea of intensive forest management as a pathway towards economically and ecologically sustainable forest management. Environmental NGOs and forest companies have been collaborating under various platforms, for instance, the Association of Ecologically Responsible Timber Companies^2^ (established in 1999 as part of WWF Global Forest and Trade Network) and the Boreal Forest Platform^3^ (established in 2015).

It is assumed that the introduction of intensive forest management should lead to a transition from ‘mining of wood’ in high ecologically valuable primary forests to intensive forestry in less ecologically valuable secondary forests. In Russia, the discursive coalition of environmental NGOs and forest companies has been enabled by these shared ideas on sustainable forest management, albeit motivated by different core beliefs, values and interests. The companies aim at sustaining the supply of wood raw materials to factories and mills to reap revenues and avoid additional or increasing production costs caused by wood mining due to increasing utilization and transportation costs, secure investments and meet market requirements on the (economic) sustainability of wood sources. For their part, the environmental NGOs focus on intensive forestry in secondary forests to reduce pressure on primary forests and enforce the conservation of biodiversity in those primary forests, including intact forest landscapes. Moreover, environmental NGOs call for transition towards intensive sustainable forest management, where ‘sustainable’ means to them biodiversity maintenance in managed forests (similar to woodland key habitats in Nordic and Baltic countries).

The need for cooperation between forest companies and environmental NGOs is driven by market demands for sustainable wood sources. The FSC standards do not directly require the intensive forest management model. However, in Russia, FSC certification indirectly contributes to the development of intensive forest management through the requirements for sustained yield and primary forest conservation:

A change in the current situation for the better is possible with the transition to the intensive forest management model... Proper use of forests... will protect intact, ecologically and socially valuable, forest areas. This will reaffirm to the buyers of our products that forest resources… are being consumed in a sustainable way; representative of a forest company (Source 2).

The environmental NGOs and forest companies both have been lobbying for the institutionalization of the principles of intensive forest management within the framework of state regulation of forestry in forest concessions since the beginning of the 2000s. In the 2010s forest depletion and a wood supply crisis became more visible in the country and state policymakers could not ignore them. As a result, the idea of intensive forest management as a solution to forest depletion became mainstream in Russian forest policy debates.

The strategy [The National Strategy for the Development of the Forest Sector] reinforces the transition to the intensive forest management model. This means that we are improving the economy of wood harvesting not by involving new forest lands in felling, but by efficiently organizing thinning and forest care; Deputy Prime Minister of the Russian Federation (Source 7).

In Russia, in the 2010s, a new facet of the discourse on intensive forest management appeared. In addition to the development of intensive forest management in state-owned forests concessions, environmental NGOs started to promote the idea of establishing forestry operations on private abandoned agricultural lands that were reforested by natural succession. This initiative is conceptualized by environmental NGOs as a shift from ‘wood mining’ in primary forests to forest farming, agroforestry or forest plantations in abandoned agricultural lands. This new facet of the discourse on intensive forest management requires a separate analysis and is therefore not addressed in this article.

### Intensive forest management discourse: Institutionalization

The combined efforts of environmental NGOs and forest companies have raised the intensive forest management discourse in the national agenda and pushed its institutionalization under state forest policy and regulation. The concept of “the intensification of forest use and reforestation” was integrated into the National Forest Policy^4^ approved by the Government in 2013. This concept was further elaborated in two national policy documents: The Concept of Intensive Forest Use and Reforestation (the Concept)^5^,^6^ and The National Strategy for the Development of the Forest Sector until 2030 (the Strategy).^7^ The Concept was elaborated by a research institute under the Russian Federal Forest Agency. The Strategy was adopted by the federal government in 2018. These policy documents secured the institutionalization of the intensive forest management discourse under state forest policy.

The Concept recognized the (economic) problem of depletion of forest resources and the existence of a wood supply crisis in Russia. The intensive forest management model with references to the experience of Nordic countries is considered in the Concept as a (rational) solution to this (economic) crisis and as a way towards sustainable forest management. The Concept defines intensive forest management as “a system of forestry and forest use in which efforts are aimed at obtaining the maximum economic efficiency of the rotation period (from reforestation to final cutting) while observing the requirements of sustained yield and conservation of biological diversity”.

The Strategy sets the overall aim of forest management as being “to achieve sustainable forest management, innovative and efficient development of the use, conservation, and reproduction of forests, ensuring faster growth of the forest sector, social and environmental safety of the country, absolute fulfilment of Russia's international forest-related obligations”. Intensive forest management is defined as one of the key tasks for achieving the aim of the Strategy. Intensive use and reproduction of forests is mentioned in the Strategy as “guaranteed provision of the economy and society with forest resources”. The Strategy recognizes that the shortage of raw wood is a risk to the forest sector, which occurs due to two reasons: the depletion of forest resources and the exclusion of intact forest landscapes from forest exploitation due to the forest certification requirements driven by environmental NGOs. The intensive use and reproduction of forests and the development of a protected areas network are mentioned as the key measures to mitigate this risk. Therefore the Strategy provides a link between the discourse on intensive forest management and the discourse on intact forest landscapes.

**Fig. 2:**
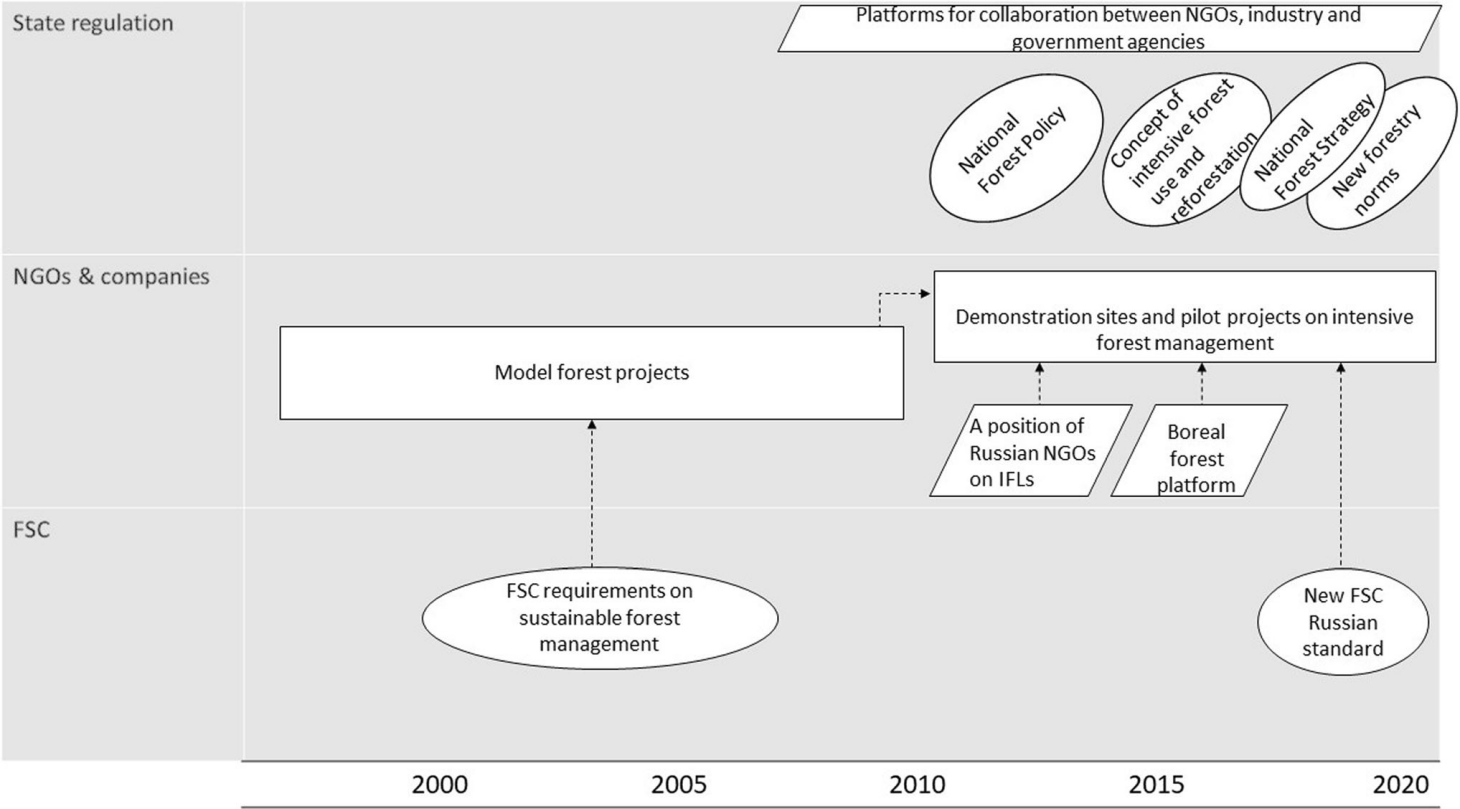
Institutionalization and materialization of the discourse on intensive forest management

In addition to policy documents, the discourse on intensive forest management has been institutionalized in a set of state regional-level forestry norms. New norms on wood harvesting, reforestation and thinning have been adopted to implement the intensive forest management model in forest concessions located in the most productive forest regions in North-West Russia and Siberia. The institutionalization of the discourse on intensive forest management in forest concessions is shown in Fig. 2.

### Intensive forest management discourse: Materialization

Two stages can be distinguished in the materialization of the discourse on intensive forest management. The first stage is the development of the idea of intensive forest management within model forests in 1997–2010 before the discourse was institutionalized within state forest policy and regulation. The second stage was characterized by the establishment of demonstration sites conducted by companies in the 2010s in parallel with the institutionalization of the discourse within state regulation.

In Russia, several model forests have been implemented aimed at transferring and adapting the Scandinavian intensive forest management model to Russian conditions (Angelstam et al. 2019). The most well known of them are the Komi Model Forest (1999–2006) run by the environmental NGO Silver Taiga Foundation and supported by the Swiss Agency for Development and Cooperation, and the Pskov Model Forest (1999–2008) managed by WWF with the support of the Swedish International Development Agency and the transnational corporation Stora Enso. Government agencies and research institutes were also among the partners of the model forest projects. Both model forests were FSC-certified to demonstrate a commitment to the international market requirements for sustainable forest management. The experience of model forests was used by the discourse coalition of environmental NGOs and forest companies to lobby for the institutionalization of intensive forest management principles under state regulation.

Since the beginning of the 2010s, several forest companies have been developing demonstration sites in their forest concessions to introduce intensive forest management based on the experience of model forests. However, despite the more than 20-year history of the discourse on intensive forest management in Russia, as well as its institutionalization under state regulation, widespread application of this approach in practice has not yet occurred (Naumov 2017; Shvarts and Shmatkov 2020). Our participant observations show a sceptical attitude among some forest professionals towards the development of the Scandinavian model of intensive forest management in Russia. The development of intensive forest management demands investments in silviculture, forest roads and infrastructure. However, complex forest tenure rights under state forest ownership and inconsistent state policy and weak law enforcement are obstacles to such investments. Forest professionals say that the lack of company motivation to invest in intensive forest management in Russia can be compared with tenants’ lack of motivation to make expensive high-quality repairs in a rented apartment where they live temporary (participant observations). Thus, a representative of the forest industry articulates his concerns as follows:

In my view, without the introduction of private ownership of the forests, proper road construction and “Scandinavian intensive forestry care” the future [of Russian forest sector] will not be bright!” (Source 4)

Furthermore, the way in which the concept of intensive forest management is being integrated into state forest policy and regulation is criticized and seen by experts as a cover-up for ‘wood mining’ in secondary forests (driven by the pulp industry):

… another “understanding” of the intensification of forest management… is based on the desire of the forest business, faced with a shortage of mature coniferous stands in leased forests, to reduce the cutting age in the forests of the Russian Federation, i.e. to meet the growing demand for coniferous wood not through its reproduction, but through the felling of middle-aged stands. Unfortunately, such proposals are today largely supported by the federal forest governance bodies (Source 1).

### The discourse on intact forest landscapes: conceptualization

Conservation of primary forest based on ideas of ‘wilderness’ and ‘intactness’ has become the mainstream in nature conservation agendas around the world since the 1980s (McCloskey and Spalding 1989; Bryant et al. 1997). In Russia, the discourse on conservation of primary forests appeared in the 1990s driven by environmental NGOs and scientists. However, until the 2000s, environmental NGOs had neither clear criteria for primary forests nor policy instruments for their conservation. The situation changed with the elaboration of the concept of intact forest landscapes and the development of forest certification as a forest conservation tool for environmental NGOs.

Although intact forest landscapes are a global concept of primary forest, this concept was first tested and implemented in Russia (Yaroshenko et al. 2001). The discourse on intact forest landscapes was conceptualized as conservation of the last large, unfragmented primary forests (Yaroshenko et al. 2001). Intact forest landscapes are considered as crucial ecosystems for biodiversity conservation, carbon sequestration, maintenance of hydrological regimes and natural ecosystem dynamic (Kobyakov et al. 2015; Potapov et al. 2017; Watson et al. 2018). In Russia, the emergence of a new NGOs-driven category of forests to be protected was negatively perceived by representatives of state agencies and the forest industry as an externally imposed and unconvincing concept (participant observations). Over time, intact forest landscapes have become a widely known, albeit not universally recognized, concept in scientific and policy debates on forest conservation:

Some attributes of natural intact forests cannot be restored after their economic development, other approaches are needed to conserve them, including their complete withdrawal from use and exclusion from the annual allowable cut (Source 1).

The first-ever maps of intact forest landscapes were developed by a group of environmental NGOs (through access to remote sensing) for European Russia in 2001, for the whole of Russia in 2003, and worldwide in 2006 (Potapov et al. 2008). Unlike previous global concepts of primary forests (McCloskey and Spalding 1989), intact forest landscapes have been mapped with sufficient accuracy to make decisions on their protection at the local level. The development of the concept of intact forest landscapes and its impact on forest conservation policy was facilitated not only by accurate maps but also by conceptualization as “high conservation value forests” (HCVFs), later “high conservation values” (HCVs) under FSC certification. Although intact forest landscapes were first explicitly recognized in the FSC Principles and Criteria in 2015 (FSC 2015), environmental NGOs in Russia had been considering them as the priority category of HCVFs (HCVs) since the early 2000s. As a result, intact forest landscapes have become the most discussed albeit conflict-ridden category of HCVFs (HCVs). Conflicts have been caused by the economic dependence of some companies on wood resources from intact forest landscapes and a lack of interest to exclude them from economically oriented forest management. In opposition to the NGO-driven discourse on intact forest landscape conservation, such companies developed a counter-discourse denying the ecological value of intact forest landscapes and considering them as over-mature forests to be cut. However, our participant observations prove that at the end of the 2010s, in Russia, debates mainly shifted from recognition versus denial of the ecological value of intact forest landscapes towards finding trade-offs for their conservation, which considered economic and social aspects. The development of intensive forest management in secondary forests is considered as a possible solution for intact forest landscapes conservation issues:

To meet the needs of the industry after the introduction of the intensive [forest management] model, it makes no sense for [forest] companies to expand to more remote areas. This makes it possible to preserve intact forest landscapes…; a researcher (Source 5).

In 2013, a group of environmental NGOs released a unified position on intact forest landscapes^8^ primarily addressed to FSC-certified companies. The position called for protection of at least half of each intact forest landscape (or the part that was located within a concession) as well as the development of intensive forest management in secondary forests as an alternative to the exploitation of intact forest landscapes. In addition to protecting intact forest landscapes through forest certification, NGOs also positioned intact forest landscapes as the priority for legally protected forests, including protected areas as well as the newly emerged concept of national forest heritage. Thus, the concept of intact forest landscapes came to have a discernible impact on policy debates on forest conservation in Russia.

The intact forest landscapes concept is also reflected in policy debates at the level of intergovernmental structures of which Russia is a part, such as the International Union for Conservation of Nature (IUCN)^9^ and the Barents Euro-Arctic Council (BEAC).^10^ Thus, in 2016, the IUCN World Conservation Congress adopted Motion 048 which calls for states, the private sector and international financial institutions to avoid loss and degradation of intact forest landscapes. In 2015, The Strategy for Protection of the Intact Forests in the Barents Region was acknowledged by the BEAC Meeting of Environment Ministers as a contribution to meet the Aichi biodiversity targets of the Convention of Biological Diversity.

### The discourse on intact forest landscapes: institutionalization

The concept of intact forest landscapes has been institutionalized under FSC forest certification standards. Initially, FSC standards did not (either globally or nationally) include measurable requirements for how much intact forest landscape should be conserved within certified forest management units. The first version of the Russian national FSC standard (adopted in 2008, with amendments in 2012 and 2015)^11^ only specified conservation of core areas of intact forests landscapes with respect to local socio-economic conditions. The turning point in the institutionalization of the intact forest landscape within the framework of FSC was the adoption of Motion 07 and Motion 65 driven by Greenpeace International at the FSC General Assembly in 2014 (FSC 2015). Motion 07 provided a clear definition of intact forest landscapes as high conservation value (FSC 2015). Motion 65 called for the development of requirements on intact forest landscapes protection within national standards. This motion implies a ‘default indicator’ that requires the protection of at least 80% of intact forest landscapes within each forest management unit if appropriate national standards are not implemented by the end of 2016. In 2017, FSC, within the framework of International Generic Indicators (IGIs), approved indicators developed for intact forest landscapes based on Motion 65. This led to the adoption of the new FSC Russian national standard in 2020 based on IGIs. The standard assumes the thresholds of 80%, 50% and 30% as the share of an intact forest landscape that needs to be conserved, depending on the measures taken by the certified company (FSC 2020). According to the standard companies perform the following: protect 80% of intact forest landscapes if the company conducts only zoning of an intact forest landscape, including the allocation of a strict conservation zone; protect 50% if the company also ensures the maintenance of biological diversity and the imitation of natural forest dynamics while conducting forestry activities; protect 30% if the company, in addition to the measures mentioned above, initiates and/or undertakes measures to assign a legal status for strict conservation zones (such as the establishment of state-led protected areas or national forest heritage sites) (FSC 2020). Despite the presence of representatives from industry who opposed these requirements for intact forest landscape conservation, they have been adopted by all three (economic, environmental and social) FSC chambers at the national level in Russia. Moreover, the new FSC Russian national standard contains a clause that recently required certified intact forest landscapes to be completely excluded from forest management starting from 2022. However, the clause is still subject to policy debate. Environmental NGO-driven institutionalization of the discourse within FSC has made significant progress both at the global level and in Russia. The requirements of FSC standards for the conservation of intact forest landscapes have been strengthened and NGOs have gained policy tools to achieve their strategic goals in primary forest conservation.

**Fig. 3:**
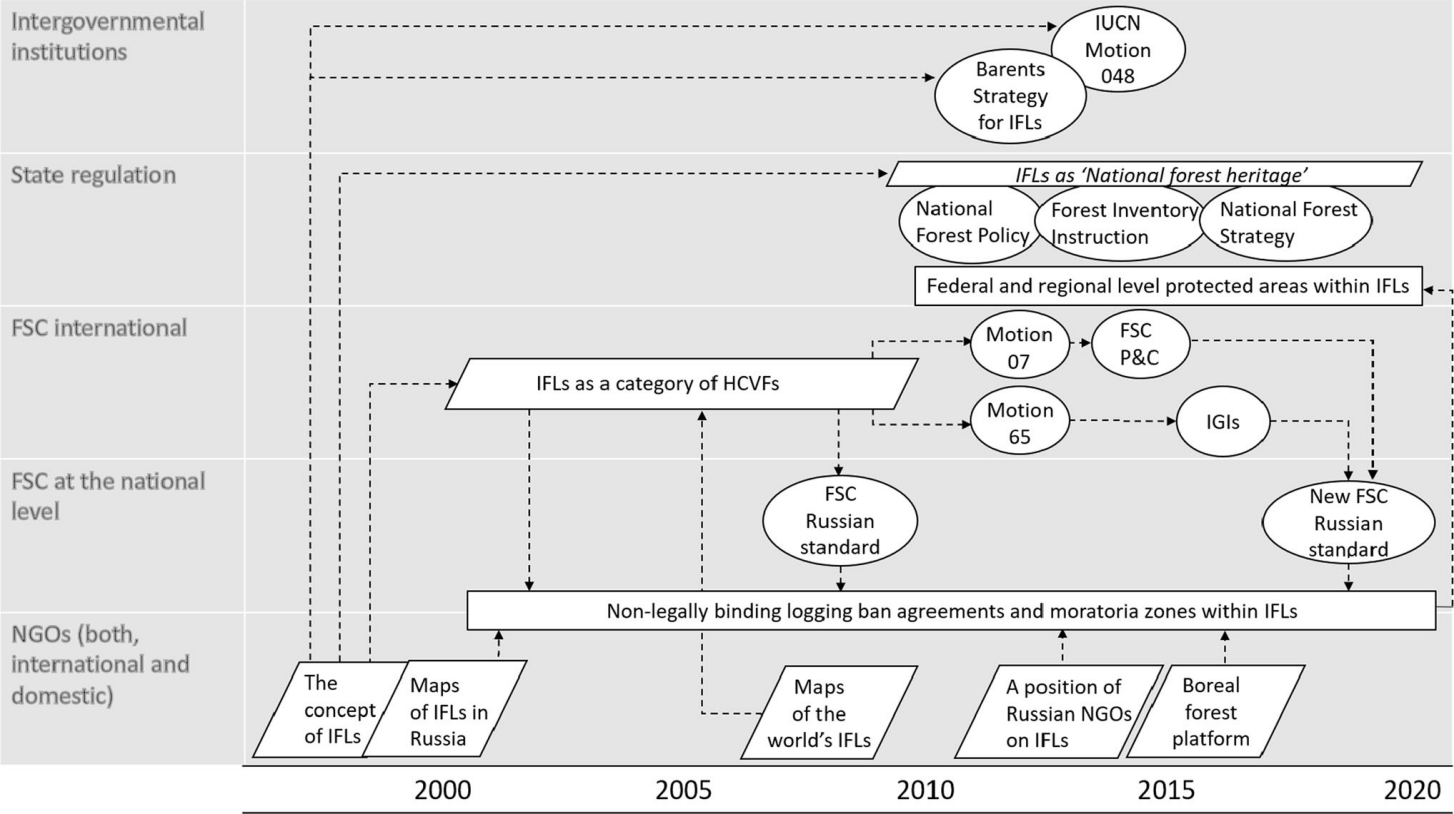
Institutionalization and materialization of the discourse on intact forest landscapes

In Russia, the institutionalization of intact forest landscapes under state regulation is fragmented and incomplete. Thus, the National Forest Policy adopted in 2013 introduced a new category of forest—national forest heritage—that should not be allocated for forest exploitation purposes. In turn, the National Forest Inventory Instruction, adopted in 2018, included intact forest landscapes as a category of national forest heritage. However, amendments^12^ to the instructions adopted in 2020 introduced an alternative definition of intact forest landscapes, which does not reflect the generally accepted concept. According to the amendments, intact forest landscapes are small fragments of habitats of relict and endemic species rather than large intact landscapes over 50 000 ha without human disturbance. Another state policy document—The National Strategy for the Development of Forest Sector—considers conservation of intact forest landscapes required by FSC standards as a threat to the development of the national forest sector. The institutionalization of the discourse on intact forest landscapes is shown in Fig. 3.

### The discourse on intact forest landscapes: Materialization

The intact forest landscapes discourse has materialized under FSC as non-legally binding logging ban agreements between NGOs and forest companies through the establishment of moratoria zones. According to FSC,^13^ as of 12.01.2020, 3 million ha of intact forest landscapes are conserved under moratoria zones (namely 1.2 million ha under permanent agreements, 1.8 million ha under temporary agreements). Considering that the total area of FSC-certified intact forest landscapes in Russia is 5.8^14^ million ha, this means that 52% of these landscapes are conserved as moratoria zones (21% under permanent agreements, 31% under temporary agreements).

Despite the use of non-state forest certification as a tool to conserve intact forest landscapes, NGOs have been lobbying for legally designated conservation status to be granted to the most valuable moratoria zones. As a result, some of the moratoria zones are considered as a basis for the creation of state protected areas (Angelstam et al. this special issue). Thus, in the 2010s, two national parks and two nature reserves (with a total area of more than 600 thousand ha) were established in North-West Russia within intact forest landscapes based on moratoria zones.^15^

## Discussion

### Discourses and discourse coalitions

Our study indicates that the analysed discourses on intensive forest management and intact forest landscapes are shaped and driven not only by non-state actors alone, but also by discourse coalitions between them. These so-called “green alliances” (Arts 2002) or “shifting coalitions” (Sotirov and Winkel 2016; Sotirov et al. 2017) between environmental NGOs and companies that is to say strategic partnership and coalitions between actors who were traditional policy opponents in the past, are increasingly discovered around the world. This finding is well in line with previous observations of issue-dependent strategic alliances in natural resource policy and governance processes, especially at the forest environmental frontier in Europe and elsewhere in the world (Sotirov and Memmler 2012; Sotirov and Winkel 2016; Sotirov et al. this special issue).

More importantly, the strategic cooperation between environmental NGOs and forest companies, and sometimes including state actors, has shaped the conceptualization, institutionalization and materialization of both discourses impacting national forest policy, regulation, and less so forestry practice in Russia. The discursive impact has helped achieve some policy and management changes in sustainable forest management and primary forest conservation despite the reluctance or opposition of other companies or state actors.

In Russia, the discursive cooperation between environmental NGOs and forest companies is stimulated as well as international market requirements for sustainable forest management and primary forest conservation, which are implemented through FSC forest certification. The impact of forest certification on sustainable forest management is still a controversial issue. On the one hand, as explained by Blumroeder et al (2019), FSC is being criticized for not being an efficient enough tool to achieve sustainable forest management and to slow down primary forest decline in Russia. On the other hand, the NGO-driven strengthening of the ecological and social requirements of FSC is contested and criticized for not considering socio-economic aspects of forest management. Such contestation has led to declining support for FSC from the industry and has increased the popularity of the less demanding industry-friendly forest certification system—like the Programme on the Endorsement of Forest Certification (PEFC)— in Russia. In this case, we are faced with a conundrum: if certification standards are very demanding, they often receive low support from industry, and as a result have low impact; if standards are less demanding, they might have higher support from industry, but also have low impact on the ground (Cashore 2012).

Discursive coalitions between environmental NGOs and forest companies are not always stable and monolithic. Some forest companies are more interested in collaboration with NGOs and FSC, while others are less so (especially when companies are unable to meet certification requirements and are trying to find alternative solutions). Thus, Henry and Tysiachniouk (2018) demonstrate the emergence of an “industry-government alliance” in Russia in which state actors support the industry’s opposition to FSC in Russia, and support the PEFC. The strengthening of FSC requirements has led to the emergence of the idea of developing an alternative state-led forest certification system in Russia, which does not relate to international certification systems. However, this idea will undoubtedly raise the issues of legitimacy and acceptance of such certification in international markets and policy.

### The analysed discourses in the context of global meta and forest discourses

National forest discourses have developed against the background of, or have even been driven by, relevant global meta-discourses, such as sustainable development and bioeconomy (Fig. 1). The intertwined Russian discourses on intensive forest management and intact forest landscapes reflect the global meta-discourse on sustainable development, where “conservation and use of natural resources are not seen as contradictory” (Pülzl et al. 2014). This global discourse believes in “producing more with less” (Arts and Buizer 2009) and “technology transfer” (Arts et al. 2010; Pülzl et al. 2014). In our study, the global idea of “producing more with less” is implemented through the policy on intensive forest management, which needs comparatively small forest areas for exploitation (compared with business as usual based on ‘wood mining’ and covering large areas). In turn, in the Russian context, “technology transfer” refers to the transfer to Russia of Scandinavian forest management concepts, ideas, approaches and technologies to develop sustainable forest management and to address forest depletion.

The intensive forest management discourse also reflects ideas from the global meta-discourse on the bioeconomy, including “export-oriented productivity” and “sustainable intensification” (Pülzl et al. 2014). In the Russian context, “export-oriented productivity” is related to economic growth via the development of an export-oriented forest sector which can reduce the national economy’s dependence on oil and gas. Although the bioeconomy is considered as a way towards sustainable development, classical forest discourses, such as sustainable forest management, biodiversity and forest conservation “take a back seat in the bioeconomy context” in the global agenda (Pülzl et al. 2014). In this regard, the analysed Russian discourse on intact forest landscapes is important in the context of the global bioeconomy narrative, and the discourse reflects the global forest discourses on “conservation in forest parks” and “forest biodiversity” (Arts et al. 2010). Even though the bioeconomy discourse is still underdeveloped in Russia, the growing political support for bioeconomy projects in parts of Europe (a key market for Russian forest products) increases the demand for Russian wood products. However, in practice, this demand can be currently met only through economic concepts, ideas, and practices: either through continued ‘wood mining’ in primary forests leading to forest depletion or through the development of intensive forest management in secondary forests based on sustained yield. Alternative discourses geared more towards ecosystem management of forests, for example, continuous cover forestry, close-to-nature or integrated forest management have not yet been conceptualized, institutionalized or materialized in Russia.

## Conclusion

Based on Hajer’s approaches to the analysis of discursive hegemony (Hajer 1995), we identified and analysed two key forest discourses driven by non-state actors—environmental NGOs and forest companies—in Russia. These discourses are related to the development of intensive forest management and the conservation of intact forest landscapes. Although they may seem mutually exclusive, our analysis shows how these discourses are closely related and positioned as two sides of the same coin.

In Russia, the discourse on the development of intensive forest management based on the Scandinavian forestry model addresses forest resource depletion and primary forest loss. Intensive forest management is conceptualized as a pathway towards sustainable forest management in secondary forests based on efficient silviculture and sustained yield in managed forests in forest concessions. This discourse concerns the transition from ‘wood mining’ in primary forests (high ecologically valuable forests) to intensive ‘tree agriculture’ in secondary forests (less ecologically valuable forests). The initially marginal idea of developing the Scandinavian intensive forestry model in Russia, driven by a discursive coalition of environmental NGOs and forest companies, has come a long way of conceptualization and institutionalization. The discourse has impacted government policymaking and has become institutionalized under state forest policy and forestry regulations. However, the idea of developing intensive forest management ‘like in Scandinavia’ has not yet been materialized in forest concessions in Russia.

The discourse on intact forest landscapes is mainly driven by environmental NGOs and these landscapes are conceptualized as the last large, unfragmented primary forests to be conserved. The discourse on intact forest landscapes is conceptualized in policy and scientific debates as crucial ecosystems for biodiversity conservation and climate change mitigation. Driven by NGOs, the concept of intact forest landscapes has been institutionalized under FSC certification as high conservation value and mentioned in state-led forest policy documents as national forest heritage sites. As part of FSC certification standards, clear requirements regarding the proportion of intact forest landscapes that should be excluded from forestry have been introduced. However, there are no similar requirements within the framework of state regulation in Russia. The concept of national forest heritage is blurred and its connection with intact forest landscapes conservation remains uncertain.

The discourse on intact forest landscapes is materialized in practice by environmental NGOs as non-legally binding moratoria zones—‘no-go zones’—in FSC-certified forest concessions. In turn, at the initiative of NGOs, some of these moratoria zones become the basis for the creation of legally designated national parks and nature reserves. Thus, the discourse on intact forest landscapes conservation, initiated by NGOs and supported by the FSC, is materialized as part of the system of state protected areas. However, the exclusion of intact forest landscapes from forestry is not compensated by the development of intensive forest management in productive and economically accessible secondary forests. Thus there is still a frontier between sustainable forest management discourses and the actual forest management practices in Russia.

## Supplementary Information

Below is the link to the electronic supplementary material.

Supplementary file1 (PDF 214 kb)

## Footnotes

1 The Food and Agriculture Organization of the United Nations.

2 https://wwf.ru/en/what-we-do/forests/gftn-russia/ (accessed on 21 May 2020).

3 https://borealforestplatform.org/en/ (accessed on 21 May 2020).

4 http://static.government.ru/media/files/41d4926bf69a218ee79f.pdf (accessed on 21 May 2020).

5 http://www.spb-niilh.ru/pdf/Rosleshoz_booklet.pdf (accessed on 21 May 2020).

6 Elaborated by Saint Petersburg Forestry Research Institute; does not have the status of a legal act.

7 http://static.government.ru/media/files/cA4eYSe0MObgNpm5hSavTdIxID77KCTL.pdf (accessed on 21 May 2020).

8 https://hcvf.ru/pub_doc/Pozicija%20nepravitelstvennyh%20prirodoohrannyh%20organizacij%20Rossii%20po%20malonarushennym%20lesnym%20territorijam.pdf (accessed on 20 May 2020).

9 The International Union for Conservation of Nature (IUCN) is a global membership union composed of both government and civil society organisations focussed on nature protection and biodiversity conservation.

10 The forum for intergovernmental cooperation between the northern regions of Norway, Sweden, Finland and Russia.

11 https://ru.fsc.org/ru-ru/cert/standarty/105310721094108010861085107210831100108510991077-108910901072108510761072108810901099 (accessed on 28 December 2020).

12 http://publication.pravo.gov.ru/Document/View/0001202011090047 (accessed on 28 December 2020).

13 http://maps.fsc.ru/ (accessed on 12 January 2021).

14 https://fscrus.nextgis.com/resource/0 (accessed on 12 January 2021).

15 https://hcvf.ru/ru/maps (accessed on 28 December 2020).
