# Supplementary material for: The forest environmental frontier in Russia: Between sustainable forest management discourses and ‘wood mining’ practice
Source: Ambio. 2021 Oct 21;50(12):2138–52. doi: 10.1007/s13280-021-01643-6 (PMC8563926; doi:10.1007/s13280-021-01643-6)
Supplement: Supplementary file 1 — Supplementary file1 (PDF 214 kb) [file 13280_2021_1643_MOESM1_ESM.pdf]

Ambio

Supplementary Information
This supplementary information has not been peer reviewed.

The forest environmental frontier in Russia: Between sustainable forest management discourses and 'wood mining' practice

Denis Dobrynin[a], Natalya Yakusheva Jarlebring[b], Irmeli Mustalahti[c], Metodi Sotirov[d], Elena Kulikova[e], Eugene Lopatin[f]

[a] Department of Geographical and Historical Studies, University of Eastern Finland, e-mail: denis.dobrynin@uef.fi (the corresponding author)

[b] Department of Forest Sciences, University of Helsinki, e-mail: natalya.yakusheva@helsinki.fi
[c] Department of Geographical and Historical Studies, University of Eastern Finland, e-mail: irmeli.mustalahti@uef.fi

[d] University of Freiburg, e-mail: metodi.sotirov@ifp.uni-freiburg.de

[e] European Forest Institute, e-mail: elena.kulikova@efi.int

[f] Natural Resources Institute Finland (Luke), e-mail: eugene.lopatin@luke.fi

Title: **Appendix 1. Examples of relevant citations characterizing the discourses**

Discourse on intensive forest management on forest concessions within state-owned forests
"The intensive model of the forest management, first of all, allows high economic efficiency of the forest sector and increase in the productivity of forests and the volume of wood harvesting to be achieved primarily in areas with a developed social and transport infrastructure, which will fully meet the demand for wood while conserving protective forests, protected areas and intact forest landscapes" (Source 1).<br><br>"The participants in scientific debates noted the presence, primarily among forest users, of another "understanding" of the intensification of forest management. This understanding is based on the desire of the forest business, faced with a shortage of mature coniferous stands in leased forests, to reduce the cutting age in the forests of the Russian Federation, i.e. to meet the growing demand for coniferous wood not through its reproduction, but through the felling of middle-aged stands. Unfortunately, such proposals are today largely supported by the federal forest governance bodies" (Source 1).<br><br>"Intensive forest management in Russia is practically undeveloped…The main barrier to the development of intensive forest management is the difficulty of changing the regulatory framework. An imperfect regulatory framework is the main deterrent", a researcher of Saint-Petersburg Forestry Research Institute (Source 2).<br><br>"Intensive forest management should be developed in secondary forests, taking into account increase in the efficiency of forest regeneration and competent management of thinning in young stands while maintaining

biodiversity (at the landscape level), including intact forest landscapes in the form of national forest heritage, with the support of the state and the creation of appropriate incentives for forest users, including through investment support and tax cuts”; (Source 3).<br><br>“It is necessary to stimulate intensive forest management in secondary forests to maximize the commercial forest yield through reforestation, forest care, multiple increases in the productivity of leased forests and the volume of investments”; Conservation Policy Director, WWF-Russia (Source 4).<br><br>“Intensive forest management - nothing will change without the voice of the forestry business”; (Source 4).<br><br>“In my view, without the introduction of private ownership of the forests, proper road construction and “Scandinavian intensive forestry” the future [of Russian forest sector] will not be bright!”; CEO of RFI Consortium Ltd., the management company of JSC Novoyeniseiskiy Wood-Chemicalcomplex (Source 4)<br><br>“The advantages of an intensive forest management model for the environment, business and society are clear"; Timber Supply Director of CJSC International Paper (Source 2).<br><br>“In Russia, forests are owned by the state and in the long term, it seems to me, the state forest ownership will remain, which implies a clear interest of the state in changing the outdated formalized regulatory framework and providing economic incentives to forest users to switch to intensive forest management”; Timber Supply Director, International Paper (Source 2).<br><br>“Intensive forest management for me is a synonym for proper forest management, the management that leads to the entire forestry cycle of operations…”; the head of the Forest Supply Planning Department, Mondi Syktyvkar LPK (Source 2).<br><br>“Unfortunately, this practice [of intensive forest management] most often does not go beyond the demonstration sites”; Deputy General Director for Forestry Planning, Metsa Forest Podporozhye (Source 2).<br><br>“If we want the forest not to be a burden on the country's budget, but to bring an adequate, comparable income, then we have to organize proper forest management. In fact, there is no alternative to intensive forest management”; a forest entrepreneur, The Forest Alternative Company (Source 2).<br><br>“Intensive forest management is a system to establish forest management in economically accessible forests, most often secondary forests, which when reused should provide a relatively cheap resource, taking into account their favourable transport accessibility”; Deputy Director of the Federal Forest Agency (Source 2).<br><br>“The strategy reinforces the transition to the intensive forest management model. This means that we are improving the economy of wood harvesting not by involving new forest lands in felling, but by efficiently organizing thinning and forest care”; Deputy Prime Minister of the Russian Federation (Source 7).
Intact forest landscapes and intact forest landscapes & intensive forest management
“Some attributes of natural intact forests cannot be restored after their economic development, other approaches are needed to conserve them, including their complete withdrawal from use and exclusion from the annual allowable cut” (Source 1).<br><br>“To meet the needs of industry after the introduction of the intensive [forest management] model, it makes no sense for enterprises to expand to more remote areas. This makes it possible to preserve intact forest landscapes…”; a researcher of Saint-Petersburg Forestry Research Institute (Source 5).

“The main reason [for the wood supply crisis] is the lack of competent forest care, conifers are overgrown with low-value deciduous trees: enterprises are forced to cut down intact forest areas and protective forests”; Conservation Policy Director, WWF-Russia (Source 4).

“A change in the current situation for the better is possible with the transition to the intensive forest management model... Proper use of forests... will protect intact, ecologically and socially valuable, forest areas. This will reaffirm to the buyers of our products that forest resources, which are very important to society, are being consumed in a proper way…”; Timber Supply Director of CJSC International Paper (Source 2).

“The exploitation of IFLs is the use of a natural resource (wild forest), the appearance and renewal of which is not associated with economic activity. The most important characteristics that determine the natural value of IFLs cannot be preserved during fragmentation and industrial use and, even more so, cannot be artificially restored. Thus, the use of IFLs is actually the use of a non-renewable natural resource, and the concept of sustainable nature management, principles and criteria of sustainable forest management are inapplicable to it”; (Source 6).

“Intensive forest management is an economic buffer for the conservation of virgin forests, intact forest landscapes”; Deputy Director of the Federal Forest Agency (Source 2).

Sources of citations:

Source 1. Materials of scientific debates “How to solve the problems of forest management in Russia? Experts’ view” organized by the Centre of Forest Ecology and Productivity of the Russian Academy of Sciences in 2015-2019 (accessed on 20 May 2020 at http://cepl.rssi.ru/category/academic-dispute/)

Source 2. Materials of the seminar on intensive forestry conducted by International Paper in 2014 in the Leningrad Region, Russia (accessed on 18 December 2020 at https://borealforestplatform.org/wp-content/uploads/2015/08/Intensivnoe_lx.pdf

Source 3. Saint-Petersburg International Forestry Forum, 2017. Materials of the forum

Source 4. Saint-Petersburg International Forestry Forum, 2018. Materials of the forum

Source 5. Saint-Petersburg International Forestry Forum, 2019. Materials of the forum

Source 6. A unified position of NGOs on intact forest landscapes in Russia (accessed on 20 May 2020 at https://hcvf.ru/pub_doc/Pozicija%20nepravitelstvennyh%20prirodoohrannyh%20organizacij%20Rossii%20po%20malonarushennym%20lesnym%20territorijam.pdf)

Source 7. The government of the Russian Federation (accessed on 22 February 2021 at http://government.ru/news/41382/)
